# Potentially traumatic events, coping strategies and associations with mental health and well-being measures among conflict-affected youth in Eastern Democratic Republic of Congo

**DOI:** 10.1186/s41256-016-0007-6

**Published:** 2016-07-21

**Authors:** Megan Cherewick, Shannon Doocy, Wietse Tol, Gilbert Burnham, Nancy Glass

**Affiliations:** 1 Department of International Health, Johns Hopkins Bloomberg School of Public Health, 615 N. Wolfe St., Baltimore, MD 21205 USA; 2 Department of Mental Health, Johns Hopkins Bloomberg School of Public Health, 624 N. Broadway, HH 863, Baltimore, MD 21205 USA; 3 Department of International Health, Johns Hopkins School of Nursing, Johns Hopkins Center for Global Health, 615 N. Wolfe St., Baltimore, MD 21205 USA

**Keywords:** Conflict, Trauma, Coping, Mental health, Resilience, Well-being

## Abstract

**Background:**

Youth in conflict and post-conflict settings are exposed to a variety of potentially-traumatic events that impact their mental health and well-being. The purposes of this study were to examine coping strategies among conflict-affected youth exposed to potentially-traumatic events and the relationship to psychological symptoms and well-being in the Democratic Republic of Congo (DRC).

**Methods:**

A total of 434 male and female youth (ages 10–15 years) completed data collection with a trained Congolese interviewer. The survey instrument included measures of exposure to potentially traumatic events, an adapted coping strategies checklist, and measures of psychosocial distress and well-being. Exploratory factor analyses was used to identify coping strategies and Hierarchical regression was used to assess how coping strategies were associated with psychological symptoms including internalizing and externalizing problems and well-being outcomes including prosocial behavior and self-esteem.

**Results:**

Exploratory Factor analysis suggested four coping strategies; problem-focused, emotion-focused, avoidance and faith-based strategies. Problem-focused coping strategies were associated with greater internalizing and externalizing problems and lower prosocial behavior in both boys and girls. However, when problem-focused strategies were used with emotion-focused coping strategies, the result was fewer internalizing problems in girls and fewer externalizing problems in boys and girls. Emotion-focused, avoidance and faith based strategies were associated with better self-esteem.

**Conclusion:**

These results suggest a complex relationship between coping strategies, psychological symptoms and well-being and contradict evidence that problem-focused strategies benefit mental health while emotion-focused strategies harm mental health, particularly in conflict and post-conflict settings. The results suggest coping flexibility, or use of multiple coping strategies may be particularly useful to improving mental health and well-being. The need for context specific understandings of coping strategies in conflict-affected populations is highlighted by the results of the study.

## Background

Youth affected by armed conflict endure a variety of traumatic stressors that impact their psychosocial health and well-being. Globally it is estimated that within the past decade, two million children lost their lives to war, six-million were severely injured or disabled, twelve million were left destitute and 300,000 children served as child soldiers [1]. Children in conflict settings are often victims of physical and sexual assault, witness violence to family and their community and are subject to chaos and destruction of their environments which can result in material deprivation, forced displacement, and lack of basic needs for food, shelter and security. The psychological impacts of war on children includes increased prevalence of post-traumatic stress disorder (PTSD), anxiety, depression [2], psychophysiological disturbances such as nightmares and trouble sleeping, fear, grief, behavioral problems [3], changes in school performance, lack of hope and personality changes [4]. Despite exposure to traumatic stress, not all children react in the same ways and it is possible for children to employ a variety of adaptive coping strategies that can limit the negative impact of trauma on mental health and well-being.

Research with youth have examined the relationship between coping strategies and mental health and well-being. How youth deal with stress can reduce effects on mental health or amplify emotional distress and associated internalizing and externalizing behaviors [5]. Lazarus (1984) originated the term coping to describe responses to stress and defined stress as a condition or feeling experienced when a person appraises an event as “exceeding his or her resources and as endangering well-being mobilize” [6]. This theory posited that the best way to measure coping was through an individual’s personal appraisal, which refers to the various ways individuals seek to modify adverse aspects of their life to minimize the internal threat of stressors. Appraisal can be primary (perception of a stressor) or secondary (evaluation of potential effectiveness and consequences of coping behaviors) [6]. Coping is effective if stress is accurately appraised and specific behavioral and cognitive strategies are used to manage, reduce or tolerate stressful events [7]. Coping strategies can have both short-term effects, for example helping to resolve the immediate stressor, and long-term effects on mental health and well-being [5].

There are multiple reasons why division of youth coping strategies into categories that are labeled beneficial or not beneficial to mental health can be problematic. Research has reached little consensus on how those strategies should be conceptually grouped and inconsistency on how beneficial or harmful particular strategies may be [8, 9]. Lazarus and Folkman’s work characterizes coping strategies as ‘emotion centered’ which seeks to regulate internal emotions and may include cognitive distraction, seeking emotional support, emotional expression and cognitive restricting and problem or ‘situation based’ which aims to change the problem or conflict [6]. Other terms used to describe coping include ‘engagement’ or ‘problem based’ coping (active/approach styles); ‘disengagement’ or ‘emotion-focused’ coping (avoidant/passive styles) [10, 11]; religious and ideological coping [12]; and primary vs. passive coping [13]. A study with war-affected children in Croatia found six distinct coping strategies including aggressive activities, problem oriented, avoidance and relaxation, emotion expression and social support seeking [4]. Research on more than 100 assessments of coping revealed over 400 different labels used to describe those categories [5]. The lack of consensus on how to distinguish coping strategies has not deterred researchers from maintaining belief that coping matters.

Defining positive adaptations or negative adaptions requires a set of assumptions about perceived desirability of that adaptation [14]. In general, scholars have argued that the use of emotion oriented coping is associated with poorer mental health and task or problem based coping is associated with better mental health outcomes [15–17]. However, researchers have also argued for a more complex understanding of coping strategies and have hypothesized why early research may have categorized emotion-focused coping as less beneficial than problem-focused coping. For example, researchers argue that emotion-focused coping may be associated with poorer mental health because of the grouping of both adaptive and maladaptive emotional strategies within the same conceptual coping strategy [9]. For example, distraction or “just trying to forget it” has been considered a maladaptive, avoidant coping strategy, however researchers have questioned whether disengagement and avoidant strategies should be considered maladaptive, particularly because in contexts of armed conflict, these strategies may be preferred by adolescents and their effect may be context dependent, requiring additional research specific to a particular context [18, 19]. In environments such as humanitarian emergencies and armed conflict, engagement (problem-focused) coping may be a less appropriate coping method than disengagement (emotion-focused) coping because youth may be powerless to actively change stressors related to the emergency and instead emotion-focused coping may be a positive strategy that is more easily accessible than problem-focused strategies [20].

Researchers caution against relying on normative judgments derived from western culture [21]. Considering the cultural context in which coping strategies are employed is essential to gain depth of meaning to motivations for employing a particular strategy and the positive or negative effects of using a particular strategy [22]. For example, qualitative case-study research with Cambodian refugees found that avoidant coping was utilized by traumatized Cambodian refugees who sought to avoid thoughts, behaviors and activities that reminded them of the past and linked this coping strategy to the history of “dishonorable events in Cambodian history” and collective shame felt by Cambodians [23]. The Cambodian belief system perceives personal bad fortunes stem from dishonorable events in a previous life and therefore led individuals to use avoidant coping strategies rather than more problem-focused strategies. Qualitative narrative research with 14 Sudanese youth refugees found that a sense of communal self was thematic in interviews and that suppression and distraction were common coping strategies [24]. Participants used distraction to avoid difficult thoughts and feelings and believed that this strategy helped “protect themselves from feelings that they feel powerless to handle” [24]. Research with Zimbabwean adolescents who had experienced trauma found that there was greater use of emotion-focused strategies such as trying to calm oneself down or express emotions to others, rather than problem solving strategies because cultural norms in Zimbabwe discourage problem solving strategies that may involve confrontation or challenging elders and instead youth favor distancing, keeping to themselves and other emotion-focused strategies that may be more characteristic of a collectivistic society versus an individualistic society [25].

How coping strategies impact mental health and well-being is complex. Coping strategies interact with different types of stressors in varying ways over the life course and have bidirectional and reciprocal reinforcing relationships with other socio cultural variables. Others have found that *coping flexibility* may help explain the impact of emotion or problem-focused strategies on outcomes and suggest that coping flexibility, or use of multiple strategies (i.e., problem and emotion-focused strategies) may lead to better outcomes [26]. Furthermore, stress and coping exist within an individual’s unique socio-cultural context. Social, cultural, economic, political and historical processes influencing the types of stress experienced in the past, present and future as well as the coping strategies utilized. In this way, coping is a reflective phenomenon and individual coping strategies cannot be analyzed separately from the context in which they are applied. In other words, understanding adaptive behaviors is context dependent, and, “what is adaptive in one context or during one developmental period may be maladaptive during another” [27, 28]. For example, research among Palestinian children found that mother’s psychological symptoms, socio-economic stress and experiences of political hardship impacted children’s coping strategies [29]. In addition, coping strategies may be influenced by age and developmental stage. For example, children’s coping strategies may shift from behavioral to cognitive strategies as they develop. As children mature they may be more apt at calming themselves down and seeking social support as compared to avoidant strategies such as trying to forget or social withdrawal which may be more prevalent among younger children [9].

Most research has focused on how coping strategies impact psychological symptoms with less examination of how coping impacts measures of well-being such as prosocial behavior, self-esteem, outlook for the future and empathy towards others. Understanding the dynamics whereby coping protects against psychopathology and promotes well-being is important to capture the full impact of coping on mental health. Documenting how conflict-affected youth cope with adversity will improve our understanding of the types of cognitive and behavioral strategies utilized by youth and their relationships with individual, family and community factors, thus informing youth-based programs in conflict and post-conflict settings.

### Conflict and coping in the Democratic Republic of Congo

In the Democratic Republic of Congo (DRC), protracted conflict has caused instability, destruction of infrastructure and resources, forced displacement and experiences of ongoing violence. It is believed that 30,000 children are child soldiers with armed groups in the DRC [30]. A recent study in eastern DRC found that 95 % of youth reported at least one traumatic event; on average, adolescents were exposed to 4.7 traumatic events, and 52 % of adolescents met the criteria for Post Traumatic Stress Disorder (PTSD) [31]. This research questioned whether disengagement (emotion-focused) coping should be construed as a maladaptive reaction to conflict and argues for a more detailed, context specific understanding of coping strategies [18]. The study found that disengagement coping behaviors, such as distraction, resignation and social withdrawal were more common among adolescents exposed to conflict as compared to engagement behaviors such as cognitive restructuring or problem solving [18]. Violence, population displacement and the destruction of health and educational institutions have exposed youth to many types of potentially traumatic events (pTEs) that impact youth mental health and well-being.

## Research aim

Research has indicated that coping strategies vary in different contexts. Accordingly, this research aims to:

1. Find a suitable factor structure based on the adapted KidCope scale to identify latent factor strategies for youth affected by armed conflict in the DRC.
2. Evaluate age and gender differences in potentially traumatic event exposure and coping strategies
3. Identify associations between coping strategies, interaction effects and internalizing and externalizing problems.
4. Identify associations between coping strategies, interaction effects and measures of well-being including self-esteem and prosocial behavior.

## Methods

### Study setting

This study took place in ten villages in the Walungu territory in South Kivu province, Eastern Democratic Republic of Congo. This territory is 50 km south of Bukavu, the capital of South Kivu and has been afflicted by war since 1999. The Walungu territory has an estimated population of 700,000 with each participating village having populations between 75 and 350 households. This study was conducted in partnership with Programme d'Appui aux Initiatives Economiques (PAIDEK), a non-profit, non-governmental Congolese microfinance organization founded in 1995 in the Democratic Republic of Congo. This study was nested within our collaborative microfinance project, Rabbits for Resilience (RFR). RFR is a pragmatic community trial to test the effectiveness of a youth-led rabbit animal husbandry microfinance program aimed at improving health, economic stability and relationships between youth, families and communities through loans of rabbits to youth [32].

### Study procedures

Ten rural villages of the Walungu Territory were selected for participation in RFR and were determined by the operational feasibility of working in this area. Within each village households were invited to participate if the household met RFR criteria (resident of village, youth in target age group, interest in animal husbandry, vulnerable children and families). Male or female children ages 10–15 in households were eligible for participation. Only one youth per household was selected at random (stratified by gender) and enrolled to complete data collection. This current analysis includes data with participants collected at six month follow up from the Rabbits for Resilience survey.

### Data collection

This study was completed with support of trained Congolese Microfinance and Research Agents. Each investigator and research agent was given face-to-face training on responsible conduct of research, completed the on-line Collaborative Institutional Training Initiative (CITI), received a training manual, and had hands-on instruction by an expert programmer on use of data collection with the iPad. After research agents pilot tested use of the iPad to collect survey data they research team determined the iPad had a number of advantages over paper based survey instruments including 1) security and reduction of study data because of automatic uploading of data to a secure server followed by deletion of data on the iPad to minimize data loss and ensure confidentiality; 2) reduction of survey time and burden for the participant; 3) improved confidentiality and respect reported by participants, evidenced by increased use of direct eye contact. Use of iPad technology in survey implementation has been well-received in low and middle income countries and has resulted in substantial cost and time savings [33]. Use of iPad technology allowed for real-time analysis of data collection from the field to examine patterns that would indicate problems with data collection such as a poorly understood item/measure or inconsistent responses. Experience during pilot tests with the survey instrument and prior experience working in these communities indicate that youth felt comfortable being interviewed by male and female team members [32, 34]. The survey instrument was developed from existing, validated assessment tools and findings from the teams prior research, as described below, and administered electronically using a designed HTML5 survey application on tablet computers (iPad) using the iOS mobile platform (Apple Inc., Cupertino CA) to ensure consistency and to allow for data to be securely stored in a password protected file on a server. All interviews were conducted by Congolese researchers fluent in French, Swahili and a local language, Mashi. Participants selected the language they preferred for the interview. Interviews were conducted in a private setting and ranged from 45 to 90 min. All participants were provided with compensation for their time equal to USD2, an amount considered appropriate after consultation with village leaders and research team members.

### Survey measures

*KidCope* is a checklist to measure coping among youth developed by Spirito (1988) [35]. We adapted the KidCope version designed for ages 7–12. This scale consists of two parts. The first part asks if youth used (yes/no) certain coping strategies. The second part of the questionnaire asks about youth perceptions of if the behavior helped (“not at all,” “a little” and “a lot”). The original KidCope includes 15 items designed to assess ten coping strategies: social withdrawal, distraction, wishful thinking, cognitive restructuring, social support, problem-solving, self-criticism, emotional regulation, resignation and blaming others. Prior to collecting quantitative data on coping strategies, qualitative research was conducted in mid-2014 and the scale was adapted to the context and to improve cultural relevance [36]. One item, “I slept to feel better” was added to the scale to represent the “resignation” strategy and an additional coping strategy. “I sang a song to feel better” was added to represent the “emotional regulation” strategy. “I prayed” was added to the scale as a separate coping strategy based on the qualitative study that indicated prayer was a very common response to coping with stress. Strategies represented by two items were coded positive for use if at least one of the two items was endorsed, a scoring method previously used with KidCope [37]. Similar to previous use in administering the KidCope, a stressful event was identified by each youth and they were asked to consider whether they used a series of coping strategies in response to the event.

*The Harvard Trauma Questionnaire* was adapted to measure potentially traumatic events (pTEs) exposure [38]. This self-report scale measures a variety of pTEs experienced in an individual’s lifetime. Exposure to pTEs was analyzed as a continuous variable (0–18 total pTE events) and categorically. The grouping of pTEs was drawn from a study with Cambodian refugees with dichotomous exposure variables for at least one event in each category: [1] material deprivation (three events: lack of food or water, lack of shelter, and ill health without access to medical care); [2] warlike conditions (one event: combat situation); [3] bodily injury (four events: torture or witnessed torture, serious injury, rape or sexual assault, other type of sexual humiliation); [4] coercion (six events: imprisonment, brainwashing, lost or kidnapped, being close to death, forced isolation, forced separation from family members); and [5] violence to others (four events: unnatural death of family member or friend, murder of family member or friend, murder of stranger, witness rape or sexual abuse) [39].

*The African Youth Psychological Assessment (AYPA)* was used to measure internalizing (depression/anxiety) and externalizing problems (aggression/hostility) and prosocial attitudes/behaviors [40]. This scale was developed through item-response theory in multiple samples of youth in sub-Saharan Africa to assess emotional and behavioral problems, somatic symptoms and pro-social behavior and has demonstrated good psychometric properties. The scale has four response categories, none = 0, sometimes = 1, often = 2 and constant = 3 and mean scores are determined for each sub-dimension (internalizing problems, externalizing problems, somatic symptoms and prosocial behaviors). The internalizing problems subscale included items such as, “I feel sad”, “I feel a lot of pain in my heart”, “I sit with my cheek in my palm” and “I have a lot of worries”. The externalizing problems subscale included items such as, “I insult friends”, “I am disobedient” “I deceive” “I am a rough person” and “I use bad language.” The prosocial attitudes and behavior subscale included items such as, “I cooperate with others”, “I play together with others”, “I help others”, and “I share food and eat with others”. All subscales have been shown to have satisfactory reliability with Cronbach’s alpha values of prosocial behaviors/attitudes (alpha = 0.72), externalizing problems (alpha = 0.83) and internalizing problems (alpha = 0.88) [40].

*The Rosenberg Self-Esteem (RSE) Scale* was used to measure self esteem, defined as, “the degree to which he holds attitudes of acceptance or rejection toward himself” [41]. The RSE is ten point scale constructed from ten dichotomous variables with questions such as “On the whole, I am satisfied with myself”, “I feel that I have a number of good qualities” and “I feel that I’m a person of worth, at least on an equal plane with others”. The RSE has demonstrated excellent internal consistency (alpha = 0.92) and test retest reliability over two weeks with correlations of 0.85–0.88 [41].

*The Bryant Empathy Index for Children and Adolescents* was adapted (ten item version) to measure empathy [42]. Bryant defines empathy as a, “vicarious emotional response to the perceived emotional experiences of others” [42]. Empathy was measured using a ten point scale constructed from dichotomous variables such as “Seeing a boy who is crying makes me feel like crying”, “I get upset when I see a girl being hurt”, “I feel upset when I see a classmate being punished unfairly”. Cronbach’s alpha coefficient ranges 0.68–0.79 and test retest reliability coefficients were found to range from 0.81–0.85 [42].

*Additional Variables* Age was measured as a continuous variable in one-year increments. Home violence and village violence measured how safe or unsafe individuals felt in their home/village in the past six months with 1 = “unsafe” and 0 = “safe”. Being a victim of an attack within the past six months was measured as 1 = “yes” and 0 = “No”. These variables were included based on input from the Congolese research team and upon analysis of baseline data.

### Compliance with ethical standards

The Johns Hopkins School of Medicine Institutional Review Board (IRB) approved this study (IRB: CIR00001977; Date: 06-23-14). A committee of respected Congolese educators at the Universite Catholique at Bukavu reviewed and approved this study as there is no formal institutional review board in South Kivu. The research team received approval to conduct the research with local partners PAIDEK by village traditional and administrative leaders. All research team members successfully completed research training on responsible conduct of research using the on-line Collaborative Institutional Training Initiative prior to their involvement in the study.

Parents/caregivers of eligible youth were provided with information about the purpose of the study, risks and benefits of participation in the study and then were asked to provide verbal informed consent for their child to participate. If a parent/caregiver consented, their child was asked for verbal assent after receiving a description on the purpose of the study and prior to beginning the interview. Human subjects protections protocol identified local community partners to refer youth to if youth reported safety issues, became distressed during interviews or required referrals for care following interviews. Research on ethical aspects of asking children about violence in resource poor settings has found that the, “overwhelming majority of children expressed relief about being able to discuss their experiences, and did not experience the interview as a traumatic event”, and that protocols including referral to local services minimizes harm to children who do become distressed during interviews [43]. Participants’ names were recorded separately from the interview questions and secured, all interviews were conducted in private and no information was shared outside the research team. All youth’s caregivers provided informed consent and all youth provided informed assent prior to data collection. The authors disclose no conflicts of interest.

### Statistical analysis

The current analysis is data collected at the six month follow up interviews from youth ages 10–15 enrolled in the Rabbits for Resilience (RFR) study. Data were analyzed using STATA Version 12 (Stata Corporation, College Station, TX). Prevalence of each type of coping strategy used was examined by gender and age. Item variance, skewness and inter-item correlations were examined prior to conducting factor analysis of the KidCope. Sample size was adequate for factor analysis considering common requirements of 5–10 subjects for every item analyzed as well as achieving high subject to item ratio (20:1) [44, 45]. Goodness-of-fit of the confirmatory factor analysis of a two factor solution were assessed using Bentler’s Comparative Fit Index (CFI), the Tucker Lewis Index (TLI) and the Root Mean Square Error of Approximation (RMSEA) [46–48]. CFI and TLI cutoff values should be greater than 0.95 and RMSEA close to 0.06 [46].

Exploratory factor analysis was used to explore dimensionality of the KidCope scale to find the smallest number of interpretable factors needed to account for correlations among items. Tetrachoric correlations were used for the dichotomous scale items and iterative principal factor analysis was used to analyze the factor structure. Due to skewness of the binary data, factor analysis of Pearson correlation matrix is less appropriate than a matrix of tetrachoric correlations [49]. The iterated principal factor estimation method uses initial estimates of communalities and iterates the solution to obtain better estimates. Due to correlations between factors, promax rotation was used to make the factors interpretable. The number of factors selected were identified based on conventional criteria: 1) Factors with eigenvalues > =1; 2) scree plot 3) factor loadings greater than or equal to 0.35 and 4) interpretation of the factor pattern 5) results from qualitative research in this context [50]. Factor scores were used to predict the score of each individual for the factor; this method maximizes the correlation of factor scores to the estimated factor [51].

Simple linear regressions were used to assess whether pTE exposure, sex and age were associated with coping strategies. Hierarchical robust regression models were fitted to examine the association of coping behaviors on the dependent psychosocial variables: internalizing problems/attitudes and externalizing problems/attitudes; and well-being outcomes Well-being outcomes included prosocial behavior, self esteem, and empathy. We included sex, age, total pTE exposure at baseline and recent stress exposure as covariates in Model 1 for each outcome. Model 2 included coping strategies and Model 3 evaluated interaction effects of coping strategies.

## Results

### Sample demographics

The final sample of 434 youths included 224 boys (51.6 %) and 210 girls (48.4 %) and the mean age was 12.8 (SD = 1.8) (Table 1). A total of 386 (89.2 %) youth were currently enrolled in school with 289 (66.5 %) enrolled in primary school and 83 (19.1 %) youth enrolled in secondary school.

**Table 1:** Demographic characteristics of youth in the study sample

Gender	*N* = 434	%
Male	210	51.6
Female	224	48.4
Age		
10	60	13.9
11	56	12.9
12	78	18.0
13	58	13.4
14	65	15.0
15	116	26.8
Mean age (SD)	12.8 (1.77)	
Enrollment in school	*N*	%
Enrolled in school	386	89.2
Not enrolled in school	47	10.9
Class level	*N*	%
Primary	289	66.5
Secondary	83	19.1
Missing	14	3.2
Village	*N*	%
Karhagala	59	13.6
Kamisimbi	33	7.6
Lurhala	45	10.4
Kahembari	65	15.0
Cagombe	42	9.7
Cahi	45	10.4
Irhaga	41	9.5
Karherwa	29	6.7
Izege	44	10.1
Cize	31	7.1
Mental health and well-being outcomes	Mean score (SD)	Range
Internalizing problems	1.25 (0.26)	1–3
Externalizing problems	1.19 (0.21)	1–2.1
Prosocial attitudes/behaviors	2.93 (0.61)	1.13–4
Self-esteem	7.58 (1.22)	1–10

**Table 2:** Potentially traumatic event exposure by gender and age

	Male	Female			Ages 10–12	Ages 13–15			Total
	Mean (SD)	Mean (SD)	B	*p*	Mean (SD)	Mean (SD)	B	*p*	Mean (SD)
pTE exposure	2.22	2.31 (SD)	0.43	0.669	1.83	2.62	3.93	0.000***	2.26 (2.1)
	*N* (%)	*N* (%)	OR	*p*	*N* (%)	*N* (%)	OR	*p*	*N* (%)
Material deprivation	134 (59.8)	135 (64.3)	1.20	0.339	121 (62.4)	147 (61.5)	0.96	0.854	269 (62.0)
Bodily Injury	45 (20.1)	41 (19.5)	0.97	0.883	28 (14.4)	58 (24.3)	1.90	0.012*	86 (19.8)
Coercion	51 (22.8)	66 (31.4)	1.55	0.043*	34 (17.5)	82 (34.3)	2.46	0.000***	117 (27.0)
Combat	18 (8.0)	18 (8.6)	1.07	0.840	8 (4.1)	27 (11.3)	2.96	0.009***	36 (8.3)
Violence to others	99 (44.2)	85 (40.5)	0.86	0.433	69 (35.6)	115 (48.1)	1.68	0.009***	184 (42.4)

*p<0.05, ***p<0.001 in boldface

### Exposure to Potentially Traumatic Events (pTE’s)

The mean number of types of potentially traumatic events (pTE’s) ever experienced were 2.31 among girls and 2.22 among boys (Table 2). Older youth, ages 13–15 experienced significantly more pTE events as compared to youth ages 10–12 (2.62 vs. 1.83, *p* < 0.001). Breaking down pTE experiences by categories revealed that for all categories except material deprivation, older youth experienced more pTE events. The most common pTE experienced was material deprivation (i.e. lack of food or water, lack of shelter or ill health without access to medical care) experienced by 62 % of the sample. In total, 27 % of the sample experienced coercion (imprisonment, brainwashing, forced isolation, forced separation from family, being kidnapped or being close to death); girls experienced more coercion than boys 31.4 % versus 22.8 % respectively, and this difference was statistically significant (*p* = 0.043). In total, 42.4 % of the sample experienced or witnessed violence to others (unnatural death of family or friend, murder of family or friend, murder of stranger, witness to rape or sexual violence) with 48.1 % of ages 13–15 and 35.6 % of ages 10–12 experiencing violence to others (*p* < 0.0001). In total, 19.8 % of the sample experienced bodily injury (torture, serious injury, rape or sexual assault or other types of sexual humiliation) with 24.3 % of youth ages 13–15 experiencing bodily injury and 14.4 % of ages 10–12 experiencing bodily injury (*p* = 0.009). The lowest type of pTE was experiences of combat with 8.3 % of the total sample, 11.3 % of ages 13–15 and 4.1 % of ages 10–12 having experienced combat (*p* = 0.009).

### KidCope factor analysis

Confirmatory factor analyses were used to assess the adequacy of factor structures suggested by previous studies using the KidCope. The only study to date that has used the KidCope in conflict settings was a study conducted by Mels et al. [18] in the DRC which found a 2 factor engagement/disengagement factor structure had reasonable reliabilities and acceptable fit for subscales [18]. In this study, the fit for the engagement and disengagement two-factor model indicated poor fit CFI = 0.683, TLI = 0.594, and RMSEA = 0.040. Because the two-factor model did not yield good fit to the current data, exploratory factor analysis was used to establish a suitable factor structure for these data. Wishful thinking and blaming self/others were dropped at the item level due to low response rate in our population (<5 %). Social withdrawal and cognitive restructuring were removed due to high cross loadings and collinearity with social support and prayer respectively. The four factors retained accounted for 81.7 % of the variability in the data. The four retained factors were defined as *problem-focused coping* including behavioral and cognitive attempts directed toward fixing the cause of a problem, *emotion-focused coping* focused on changing one’s own emotions to feel better through self-regulation, social support seeking and rest, *avoidant coping* strategies including attempts to “just forget it” or distract oneself by playing a game or engaging in another activity, and *faith based coping* including use of prayer in response to a stressor (Table 3). Avoidance and problem-focused strategies were correlated in a small but statistically significant manner (Corr = 0.15; *p* = 0.0020), and avoidance and faith based strategies had a small but statistically significant negative correlation (Corr = −0.12; *p* = 0.0119).

**Table 3:** Oblique promax rotated factor loadings and eigenvalues

Adapted KidCope item	Coping category (Spirito et.al 1998)	Problem-focused	Emotion focused	Avoidance	Faith
Do something else	Distraction	−0.27	0.03	**0.76**	−0.07
Try to Forget it					
Try to fix the problem by thinking of answers	Problem Solving	**0.82**	0.05	−0.20	−0.05
Try to fix the problem by doing something					
Try to calm yourself down	Emotional Regulation	0.20	**0.71**	0.07	0.06
Sing a song to calm down^a^					
Try to feel better by spending time with family and friends	Seeking Social Support	0.20	**0.39**	0.04	−0.08
Do nothing because problem could not be fixed	Resignation	−0.29	**0.46**	−0.27	−0.29
Just go to sleep^a^					
Prayer to feel better^a^	Prayer^a^	−0.30	0.01	−0.28	**0.50**

^a^Additional Items based on qualitative research

Factor loadings > 0.35 in boldface

Analysis of coping strategy by age and sex indicated avoidant and emotion-focused strategies were the most commonly utilized strategies in our sample (Table 4). In addition, older youth, ages 13–15, used more emotion-focused strategies than younger youth ages 10–12. There was no significant difference in use of any coping strategies by sex in the bivariate regression analysis, however there was a marginal significant difference in problem-focused coping strategies with boys using more of this type of strategy than girls (*p* = 0.076).

**Table 4:** Coping strategy use by age and sex

	Mean (SD)				Mean (SD)				Mean (SD)
	Male *N* = 224	Female *N* = 210			Ages 10–12 *N* = 194	Ages 13–15 *N* = 239			Total *N* = 434
Coping strategy	Mean (SD)	Mean (SD)	t	*p*	Mean (SD)	Mean (SD)	t	*p*	Mean (SD)
Problem-focused	0.15 (0.35)	0.09 (0.28)	−1.78	0.076	0.10 (0.29)	0.13 (0.33)	1.10	0.270	0.12 (0.32)
Emotion-focused	0.14 (0.21)	0.16 (0.25)	0.83	0.406	0.12 (0.20)	0.18 (0.24)	2.77	0.006**	0.15 (0.23)
Avoidance	0.18 (0.39)	0.23 (0.40)	1.19	0.237	0.19 (0.38)	0.22 (0.41)	0.88	0.380	0.20 (0.39)
Faith	0.09 (0.30)	0.10 (0.30)	1.32	0.188	0.10 (0.29)	0.09 (0.31)	−0.52	0.606	0.10 (0.30)
Total	0.55 (0.62)	0.58 (0.65)	0.45	0.656	0.51 (0.60)	0.62 (0.65)	1.85	0.065	0.56 (0.63)

**p<0.01 in boldface

**Table 5:** Simple linear regressions of coping strategy use on potentially traumatic event exposure

	Problem-focused		Emotion-focused		Avoidance		Faith	
	B (SE)	*P*	B (SE)	*P*	B (SE)	*P*	B (SE)	*P*
Total pTE	−0.02 (0.01)	0.043*	0.01 (0.01)	0.016*	−0.01 (0.01)	0.139	−0.00 (0.01)	0.605
Material deprivation	−0.03 (0.03)	0.282	0.03 (0.02)	0.125	−0.01 (0.04)	0.773	0.03 (0.03)	0.330
Bodily injury	−0.09 (0.04)	0.029*	0.01 (0.03)	0.655	−0.08 (0.05)	0.089	0.01 (0.04)	0.768
Combat	−0.09 (0.06)	0.113	0.05 (0.04)	0.255	−0.08 (0.07)	0.251	−0.06 (0.05)	0.221
Coercion	−0.04 (0.03)	0.253	0.06 (0.02)	0.021*	−0.06 (0.04)	0.179	−0.01 (0.03)	0.729
Violence to others	−0.05 (0.03)	0.104	0.05 (0.02)	0.025*	−0.09 (0.04)	0.016*	−0.02 (0.03)	0.476

*p<0.05 in boldface

Simple linear regression of coping strategy use on total pTE exposure and pTE type revealed statistically significant associations (Table 5.). Total pTE experiences were negatively associated with problem-focused strategies (B = −0.02; *p* = 0.043) and positively associated with emotion-focused strategies (B = 0.01; *p* = 0.016) suggesting that as cumulative pTE exposure increases, youth tend to use problem-focused strategies less and emotion-focused strategies more. Experience of bodily injury reduced use of problem-focused strategies (*p* = 0.029). Experiencing coercion increased use of emotion-focused strategies (B = 0.06; *p* = 0.021). Witnessing violence to others increased use of emotion-focused strategies (B = 0.05; *p* = 0.025) and reduced use of avoidance (B = −0.09; *p* = 0.016). Exposure to pTE, by total experiences or pTE type, did not have any significant association with use of the faith-based coping strategy.

The results of regression analysis on internalizing problems are presented separately for males and females in Table 6. Model 3 best explained variance in internalizing problems for girls (15.4 %) and Model 2 best explained variance in internalizing problems in boys (25.1 %). In older boys there were fewer internalizing problems reported (β = −0.15, *p* = 0.016). Total pTE exposure (β = 0.26, *p* < 0.0001) and being a victim of an attack (β = 0.18, *p* = 0.001) was associated with increased internalizing problems in boys and belief their home was not safe was associated with increased internalizing problems in both boys (β = 0.23, *p* < 0.0001) and girls (β = 0.19, *p* < 0.005).

**Table 6:** Multivariable hierarchical regressions internalizing problems on independent variables

		Boys^a^				Girls^b^			
Model		b	SE	β	*p*	b	SE	β	*p*
M1	Age	−0.015	0.007	−0.139	0.030*	0.009	0.008	0.083	0.232
	Total pTEs	0.020	0.006	0.223	0.002**	0.011	0.006	0.118	0.060
	Attack Victim	0.126	0.038	0.176	0.001**	0.094	0.062	0.113	0.131
	Home Violence	0.128	0.039	0.225	0.001**	0.090	0.032	0.190	0.005**
	Village Violence	−0.067	0.028	−0.139	0.017*	0.012	0.035	0.024	0.735
	Constant	1.347	0.089		0.000	1.043	0.094		0.000
M2	Age	−0.015	0.006	−0.146	0.016*	0.009	0.008	0.081	0.242
	Total pTEs	0.024	0.006	0.263	0.000***	0.012	0.006	0.127	0.040*
	Attack Victim	0.130	0.040	0.180	0.001*	0.086	0.059	0.105	0.145
	Home Violence	0.133	0.036	0.233	0.000***	0.095	0.032	0.200	0.003**
	Village Violence	−0.056	0.028	−0.116	0.049*	0.013	0.035	0.026	0.715
	Problem-focused	0.169	0.038	0.310	0.000***	0.111	0.053	0.163	0.040*
	Emotion-focused	−0.092	0.045	−0.101	0.041*	−0.027	0.049	−0.034	0.586
	Avoidance	0.001	0.028	0.001	0.981	−0.053	0.030	−0.113	0.076
	Faith	−0.035	0.036	−0.057	0.330	0.007	0.041	0.011	0.867
	Constant	1.338	0.085		0.000	1.049	0.094		0.000
M3	Age	−0.015	0.006	−0.142	0.020*	0.011	0.007	0.101	0.133
	Total pTEs	0.024	0.006	0.258	0.000***	0.012	0.006	0.122	0.052
	Attack Victim	0.133	0.040	0.185	0.001**	0.080	0.058	0.097	0.167
	Home Violence	0.132	0.035	0.231	0.000***	0.091	0.032	0.191	0.005**
	Village Violence	−0.055	0.028	−0.115	0.050	0.022	0.033	0.046	0.507
	Problem-focused	0.200	0.050	0.368	0.000***	0.239	0.062	0.353	0.000***
	Emotion-focused	−0.074	0.045	−0.082	0.100	0.038	0.050	0.050	0.443
	Avoidance	0.003	0.028	0.007	0.911	−0.055	0.029	−0.119	**0.055**
	Faith	−0.027	0.036	−0.044	0.457	0.026	0.042	0.041	0.538
	ProblemxEmotion	−0.214	0.146	−0.094	0.144	−0.463	0.118	−0.306	0.000***
	Constant	1.328	0.085		0.000	1.008	0.089		0.000

Note: *SE* Robust standard errors

^a^ *R* ^2^ = 0.1355 for step 1, *p* = 0.0000; *R* ^2^ = 0.2458 for step 2, *p* = 0.0001; *R* ^2^ = 0.2507 for step 3, *p* = 0.1437

^b^ *R* ^2^ = 0.0747 for step 1, *p* = 0.0064; *R* ^2^ = 0.1076 for step 2, *p* = 0.034; *R* ^2^ = 0.1537 for step 3, *p* = 0.0001

*p<0.05, **p<0.01, ***p<0.001, *p*<0.10 in boldface

Problem-focused coping was significantly associated with increased internalizing problems in both boys (β =0.31; *p* < 0.0001) and girls (β =0.35, *p* < 0.0001). When the interaction term of problem-focused coping with emotion-focused coping was included in model 3 in the hierarchical regression analysis for girls, it was found that the interaction effect significantly reduced internalizing problems in girls (β = −0.31; *p* < 0.0001). Use of emotion-focused coping strategies was associated with reduced internalizing problems in boys (β = −0.10; *p* = <0.041), however when the model introduces the interaction term of problem-focused*emotion-focused coping, this result loses significance. Use of the avoidance coping strategy was marginally significant in reducing internalizing symptoms in girls (β = −0.12; *p* = <0.055).

The results of hierarchical regressions on externalizing problems indicated model 3 was the best fit for explaining the variance in externalizing symptoms in boys (8.5 %) and girls (10.4 %) (Table 7). Problem-focused coping increased externalizing problems in both boys (β =0.34; *p* = <0.0001) and girls (β =0.37; *p* = <0.001). The interaction effect of problem-focused coping with emotion-focused coping was associated with decreased externalizing problems in boys (β = −0.17; *p* = <0.047) and girls (β = −0.24; *p* = <0.009).

**Table 7:** Multivariable hierarchical regressions of externalizing problems on independent variables

		Boys^a^				Girls^b^			
Model		b	SE	β	*p*	b	SE	β	*p*
M1	Age	−0.003	0.005	−0.033	0.608	0.002	0.005	0.031	0.647
	Total pTEs	−0.003	0.004	−0.055	0.336	0.003	0.005	0.043	0.533
	Attack Victim	0.015	0.041	0.026	0.717	0.068	0.043	0.121	0.113
	Home Violence	0.023	0.035	0.052	0.514	0.006	0.021	0.018	0.760
	Village Violence	−0.032	0.026	−0.088	0.226	−0.008	0.022	−0.022	0.728
	Constant	1.188	0.065		0.000	1.101	0.067		0.000
M2	Age	−0.004	0.005	−0.051	0.393	0.002	0.005	0.023	0.731
	Total pTEs	−0.002	0.004	−0.032	0.595	0.003	0.005	0.042	0.549
	Attack Victim	0.013	0.042	0.023	0.757	0.067	0.039	0.119	0.083
	Home Violence	0.023	0.032	0.053	0.482	0.012	0.020	0.035	0.547
	Village Violence	−0.023	0.027	−0.064	0.390	−0.008	0.022	−0.023	0.720
	Problem-focused	0.094	0.027	0.231	0.001**	0.113	0.046	0.219	0.016*
	Emotion-focused	0.040	0.044	0.059	0.368	0.016	0.041	0.028	0.701
	Avoidance	−0.014	0.022	−0.040	0.511	−0.046	0.024	−0.131	0.058
	Faith	−0.003	0.029	−0.007	0.915	−0.021	0.028	−0.045	0.457
	Constant	1.186	0.061		0.000	1.109	0.066		0.000
M3	Age	−0.003	0.005	−0.043	0.468	0.003	0.005	0.032	0.629
	Total pTEs	−0.003	0.004	−0.042	0.486	0.003	0.005	0.038	0.596
	Attack Victim	0.018	0.042	0.031	0.672	0.063	0.038	0.112	0.099
	Home Violence	0.022	0.032	0.051	0.485	0.009	0.020	0.027	0.633
	Village Violence	−0.023	0.026	−0.065	0.375	−0.003	0.021	−0.007	0.903
	Problem-focused	0.137	0.034	0.339	0.000**	0.190	0.056	0.369	0.001**
	Emotion-focused	0.063	0.045	0.093	0.168	0.047	0.042	0.082	0.262
	Avoidance	−0.011	0.022	−0.031	0.616	−0.045	0.023	−0.129	0.055
	Faith	0.007	0.029	0.016	0.795	−0.012	0.028	−0.025	0.679
	ProblemxEmotion	−0.298	0.149	−0.174	0.047*	−0.303	0.115	−0.241	0.009**
	Constant	1.172	0.061		0.000	1.093	0.063		0.000

Note: *SE* Robust standard errors

^a^ *R* ^2^ = 0.0143 for step 1, *p* = 0.6141; *R* ^2^ = 0.0678 for step 2, *p* = 0.0138; *R* ^2^ = 0.0846 for step 3, *p* = 0.0471

^b^ *R* ^2^ = 0.0188 for step 1, *p* = 0.5419; *R* ^2^ = 0.0734 for step 2, *p* = 0.0967; *R* ^2^ = 0.1043 for step 3, *p* = 0.0093

*p<0.05, **p<0.01 in boldface

Regressions on prosocial behavior revealed Model 2 fit best for both boys and girls and no interaction terms between coping strategies were significant (Table 8). Model 2 explained 12.1 % of the total variance in prosocial behavior for boys and 12.7 % of the total variance in prosocial behavior for girls. Problem-focused coping reduced prosocial behavior scores in both boys (β = −0.32; *p* = <0.0001) and girls (β = −0.24; *p* = <0.0001). For girls, feeling that they were not safe from violence at home decreased pro-social behavior (β = −0.18; *p* = 0.008).

**Table 8:** Multivariable hierarchical regressions of prosocial behavior on independent variables

		Boys^a^				Girls^b^			
Model		b	SE	β	*p*	b	SE	β	*p*
M1	Age	0.023	0.021	0.072	0.271	0.033	0.024	0.097	0.175
	Total pTEs	−0.010	0.016	−0.038	0.523	0.019	0.021	0.064	0.372
	Attack Victim	0.161	0.135	0.073	0.234	0.054	0.172	0.023	0.753
	Home Violence	−0.016	0.104	−0.010	0.874	−0.228	0.100	−0.157	0.023*
	Village Violence	0.001	0.113	0.001	0.990	0.102	0.101	0.071	0.311
	Constant	2.640	0.274		0.000	2.460	0.295		0.000
M2	Age	0.028	0.021	0.089	0.169	0.038	0.023	0.111	0.106
	Total pTEs	−0.020	0.015	−0.077	0.194	0.017	0.021	0.056	0.429
	Attack Victim	0.160	0.135	0.073	0.237	0.029	0.155	0.012	0.850
	Home Violence	−0.024	0.106	−0.014	0.822	−0.264	0.099	−0.181	0.008**
	Village Violence	−0.043	0.114	−0.029	0.709	0.097	0.095	0.067	0.310
	Problem-focused	−0.523	0.090	−0.322	0.000***	−0.509	0.121	−0.243	0.000***
	Emotion-focused	−0.016	0.161	−0.006	0.922	−0.112	0.162	−0.046	0.490
	Avoidance	0.007	0.084	0.005	0.936	0.012	0.091	0.008	0.897
	Faith	0.131	0.108	0.070	0.226	0.210	0.119	0.107	**0.080**
	Constant	2.669	0.266		0.000	2.453	0.283		0.000

Note: *SE* Robust standard errors

^a^ *R* ^2^ = 0.017 for step 1, *p* = 0.6366; *R* ^2^ = 0.1205 for step 2, *p* = 0.0000

^b^ *R* ^2^ = 0.0481 for step 1, *p* = 0.0402; *R* ^2^ = 0.1273 for step 2, *p* = 0.0002

*p<0.05, **p<0.01, ***p<0.001, *p*<0.10 in boldface

Model 2 fit best for both boys and girls and explained 17.1 % of the variance in esteem for boys and 14.3 % of the variance in self-esteem for girls (Table 9). For boys, emotion-focused coping and faith based coping increased self-esteem (emotion: β = 0.16; *p* = 0.002; faith: β = 0.15; *p* = 0.018). Having been the victim of an attack and belief that their village was not safe from violence decreased self-esteem in boys (β = −0.24; *p* = 0.001) and (β = −0.18; *p* < 0.008) respectively; and problem-focused coping decreased self-esteem for boys (β = −0.12; *p* = 0.026). For girls, avoidance increased self-esteem (β =0.18; *p* = 0.005) and faith based reached marginal significance at increasing self-esteem (β = 0.14; *p* = 0.051). Girls who felt their village was not safe had lower self-esteem (β =0.28; *p* = <0.0001).

**Table 9:** Multivariable hierarchical regressions of self-esteem on independent variables

		Boys^a^				Girls^b^			
Model		b	SE	β	*p*	b	SE	β	*p*
M1	Age	0.027	0.033	0.067	0.410	−0.032	0.035	−0.059	0.354
	Total pTEs	0.030	0.029	−0.239	0.311	−0.010	0.035	−0.021	0.779
	Attack Victim	−0.907	0.273	0.023	0.001**	0.019	0.253	0.005	0.940
	Home Violence	0.068	0.186	−0.199	0.714	0.045	0.153	0.019	0.769
	Village Violence	−0.510	0.179	0.067	0.005**	−0.696	0.167	−0.293	0.000***
	Constant	7.502	0.433		0.000	8.188	0.433		0.000
M2	Age	0.013	0.033	0.024	0.690	−0.044	0.035	−0.080	0.218
	Total pTEs	0.020	0.028	0.046	0.464	0.003	0.037	0.007	0.927
	Attack Victim	−0.912	0.259	−0.241	0.001**	0.106	0.258	0.027	0.680
	Home Violence	0.031	0.189	0.010	0.871	0.044	0.150	0.018	0.770
	Village Violence	−0.467	0.175	−0.182	0.008**	−0.654	0.162	−0.275	0.000***
	Problem-focused	−0.338	0.151	−0.123	0.026*	0.218	0.211	0.062	0.304
	Emotion-focused	0.752	0.240	0.163	0.002**	0.184	0.290	0.046	0.527
	Avoidance	0.181	0.147	0.073	0.220	0.419	0.146	0.176	0.005**
	Faith	0.496	0.207	0.154	0.018*	0.446	0.227	0.138	**0.051**
	Constant	7.568	0.423		0.000	8.091	0.438		0.000

Note: *SE* Robust standard errors

^a^ *R* ^2^ = 0.1076 for step 1, *p* = 0.0008; *R* ^2^ = 0.1709 for step 2, *p* = 0.0001

^b^ *R* ^2^ = 0.0900 for step 1, *p* = 0.0024; *R* ^2^ = 0.1432 for step 2, *p* = 0.0130

*p<0.05, **p<0.01, ***p<0.001, *p*<0.10 in boldface

## Discussion

### Coping strategies

The purpose of this study was to explore youth coping strategies and to examine associations between coping strategies and mental health and well-being outcomes in Eastern DRC. Research has called for more detailed exploration of coping strategies beyond the original engagement disengagement two factor structure originally proposed for the KidCope [52], and building on original conceptualizations of the effectiveness of problem-focused vs. emotion-focused coping strategies. The critique on this original coping strategy dichotomy is driven by the hypothesis that among trauma-affected youth, certain adaptive coping strategies such as distraction and avoidance that were originally conceptualized as maladaptive, may actually be positive adaptations in some cultures and in the context of humanitarian settings.

Factor analysis in our data revealed four distinct types of coping strategies: problem-focused, emotion-focused, avoidant and faith-based strategies. In our sample, emotion-focused and avoidance coping strategies were the most frequently reported strategies used by both male and female children. Children exposed to higher levels of pTEs were less likely to use problem-focused coping less and more likely to use emotion-focused coping. This finding is supported by previous research which indicates that problem-focused coping may be more prevalent in situations where youth have more control over their stressors and in contrast, may not be an adaptive strategy in uncontrollable situations [20, 53].

### Problem-focused coping

Problem-focused coping, which is usually perceived as beneficial actually worsened internalizing and externalizing symptoms and reduced prosocial behaviors in our sample. This is likely due to the inability of youth to directly “solve” the source of their trauma whether it was victimization or witnessing violence or material deprivation. Previous research in conflict settings supports this finding. For example, among Israeli children exposed to scud missile attacks it was found that, “persisting in problem-focused coping in a situation that cannot be changed can lead to undesirable consequences” [54]. Research among Palestinian youth found that active coping was not effective in protecting children’s mental health [29]. Similarly, Elklit et al. [55] found that problem-focused and avoidant coping strategies were related to higher levels of PTSD among trauma-affected youth in Bosnia and noted that the inability to impact life decisions may explain this finding [55]. Another study among Bosnian adolescents found that engagement coping strategies increased PTSD symptoms, whereas disengagement coping strategies were associated with fewer PTSD symptoms [19]. In the context of conflict and other humanitarian contexts, problem-focused coping as a strategy used alone may worsen internalizing and externalizing problems and reduce self-esteem and prosocial behavior. Research suggests that without effective emotional regulation, trauma affected children may exhibit increased aggressive behavior, a form of externalizing behavior [56]. It is also plausible that some of the problem-focused strategies youth employ, such as stealing to reduce economic stress or consuming alcohol to reduce emotional stress, may be harmful. Problem-focused coping strategies may add additional stress if the stressors the youth are trying to “fix” cannot be changed. Interestingly, problem-focused coping was associated with lower prosocial behavior scores in both girls and boys. This finding suggests that problem-focused coping may involve maladaptive strategies used in isolation instead of strategies that utilize social support to achieve objectives.

### Avoidant coping

Avoidant coping strategies that seek to “just forget it” or distract oneself may in the short-term be effective in reducing psychological distress in contexts of ongoing conflict with profound limitations of an individual to engage with or “fix” their stressor. For example a study with Sudanese refugees found that distancing or avoidance coping in the context of chronic stress might promote positive adaptation [57]. Use of avoidant coping may foster recovery from traumatic stress by allowing youth to distance themselves and engage in activities that help recoup lost resources [58]. Two studies with refugee youth from Vietnam and Sudan found that youth prefer not to talk about experiences of traumatic events and therefore distraction was a more commonly employed coping strategy [24, 59].

In this study, avoidant coping reduced internalizing and externalizing problems in girls, but also resulted in lower empathy in girls. No change in outcome measures was observed in boys using avoidant coping. Therefore, for girls, avoidant coping is effective in reducing psychological symptoms of internalizing and externalizing problems on the one hand, but negatively impacts the well-being measure of empathy on the other. Similar to the results found with problem-focused coping, use of avoidant coping may affect different outcomes along different paths. It is conceivable that avoidant coping strategies may limit the types of social interactions and bonds that girls form and thus negatively impact emotional connections to others and result in lower empathy for others in the community. Some research suggests that avoidant coping strategies may be more adaptive in the short term but less adaptive in the long term and consideration of adaptive trajectories in coping warrants further research [60–62].

### Emotion-focused coping

Emotion-focused strategies seek to manage emotional distress and can include disengaging from emotions, distraction, and seeking emotional support [7]. Emotion-focused coping, a strategy preferred by youth in this study and in other studies in conflict-affected contexts, may be a positive adaptive response to stress. In this study, youth ages 13–15 used more emotion-focused coping than ages 10–12 which is consistent with previous research that indicates as children develop, cortical function increases and coping repertoire shifts from behavioral to cognitive strategies [8]. Among boys, emotion-focused coping increased self-esteem. It is plausible that boys who are able to process their emotions effectively feel a greater sense of self-worth and therefore have higher self-esteem. Greater use of emotion-focused coping, particularly use of social support seeking to regulate emotions, may provide enhanced social relationships and greater closeness with peers, family and the community.

Hobfall’s Conservation of Resources theoretical model (COR) theorizes that individuals’ strive to retain, protect and build resources and that what is threatening to them is the potential or actual loss of valued resources' [63]. After people experience potentially traumatic events, they are at risk for a loss of material, social and psychological resources and with each resource loss, additional loss can occur creating a spiral of loss that can negatively impact mental health [63]. Some research suggests that emotion-focused coping may reduce stress and provide safety or “conservation of resources”, particularly in humanitarian contexts with ongoing conflict. In this way, emotion-focused coping allows youth to have control over emotional resources that can be particularly important when youth are facing resource loss at the individual, family and community level as a result of conflict. Emotion-focused coping may also be particularly effective when used in conjunction with other coping strategies (discussed below).

### Faith Based coping

Research indicates that when faced with stress, people rely on religion as a coping strategy and this strategy has been assessed as protective in cross-sectional studies, albeit with mixed evidence [64]. In this study, faith-based coping was marginally significant in girls at the *p* = 0.10 cutoff level with faith associated with better prosocial behavior (*p* = 0.80) and higher self-esteem (*p* = 0.51). These results support findings from other studies. Faith coping was associated with lower anti-social behavior and depressive symptoms among adolescent girls in the occupied Palestinian territory [65]. Religiosity was associated with lower PTSD symptoms in Bosnian and Croatian adolescents [66] and lower psychological symptoms in former Ugandan child soldiers [67]. Research suggests that positive religious coping may be linked to believing there is meaning in life, seeking support from religious community and religious forgiving, whereas negative religious coping may include reappraisal of God’s powers and spiritual discontent [68]. Use of faith based coping may also overlap with other important factors such as availability of social support systems and the degree to which youth access community resources via institutions such as the church and religious events. Research with Sudanese refugees found that participants used their belief in God as a form of emotional support [69]. Furthermore the study found that the refugee’s faith promoted social interaction through church and these interactions provided social, informational and material support [69]. More research is needed to better understand faith based coping strategies as there are conflicting results indicating that religious coping can potentially both positively and negatively affect mental health. Some research suggests that religious coping is linked with fewer symptoms of psychological distress, however other studies among conflict-affected youth found that religious coping worsened depression and anxiety symptoms among adolescents from the Gaza Strip [12]. This finding highlights that not all religious coping may impact mental health in the same way and faith-based coping strategies may be specific to cultural context and/or an individual’s conceptualization/belief in a particular religion.

### Coping flexibility and interaction effects

Interestingly, research has suggested that coping flexibility, use of multiple strategies (not necessarily at the same time) or effectively modifying a coping strategy according to the stressors present in a situation is key to understanding the impacts on psychological distress and may be more beneficial than any one strategy alone [70, 71]. Children who can adapt their coping strategies to specific stressors and are flexible in their use of coping strategies have better outcomes than children who rely solely on one type of strategy [26]. However, very little research has focused on how coping strategies interact with one another to impact outcomes [17]. Effectiveness of coping flexibility may also be dependent on culture. A meta analysis by Cheng and Chan (2014) from 11 cultural regions, found that coping flexibility was more effective in cultures that were less individualistic and more collective in how they viewed their situation [71]. The authors argue that in more individualistic societies, importance placed on autonomy leads to valuing of self consistency rather than flexibility in responses to situational demands. In contrast, countries with lower levels of individualism place greater importance on relationship between individuals and their environments and emphasize interrelated nature of existence and the persistence state of flux and change that supports situational behavior and flexibility [71].

In this study, problem-focused strategies combined with emotion-focused strategies reduced internalizing problems in girls and externalizing problems in boys and girls. This finding suggests that coping strategy flexibility may provide an opportunity for problem-focused strategies to be effective. It is also possible that problem-focused and emotion-focused coping strategies are not mutually exclusive and can overlap in their functional achievement of stress reduction and well-being. For example, trying to fix a problem can also serve to calm a person down [5]. Furthermore emotion-focused coping may be used with problem-focused coping in a cyclical and synergistic dynamic whereby emotional strength gained from emotion-focused coping provides energy for subsequent problem-focused strategies [5, 58]. Without use of emotion-focused coping, youth may lack the social support require to make problem-focused strategies a successful adaptation to stress [72].

### Implications for interventions

Results from this study are important to intervention planning and implementation with young adolescents in humanitarian settings. The ability to deal with stressors whether they are traumatic life events or every day stress is critical for healthy functioning. Studies indicate that having a larger repertoire of coping skills can buffer the effect of traumatic stress on psychological health [73]. These results indicate that problem-focused coping, when used alone are associated with increased internalizing and externalizing problems, but when used with emotion-focused coping strategies reduce the frequency of these problems. This research supports previous findings that rather than focusing on improving a particular coping strategy, engagement of multiple or groups of strategies may be most efficacious as an adaptive response to stress. Interventions that can support multiple strategies by teaching additional coping skills and/or including external factors that may support use of these strategies such as including parents, teachers and community members in intervention approaches may be particularly useful. Furthermore, interventions that focus on improving executive function or the ability to consciously plan responses and choose strategies after assessment of a stressor, may allow youth exposed to adversity to better select the most effective strategy from their coping repertoire.

Interventions have been developed to enhance coping skills in youth. Many current interventions include a focus on teaching problem solving coping skills that teach young people steps to approach conflict in a positive way. Research indicates that even in young children (ages 6–9), increased use of emotion-focused coping in response to uncontrollable stressors reduced behavioral and emotional problems [26]. Additional research has shown that emotion-focused strategies may lead to better psychological outcomes when compared to children who rely solely on problem-focused strategies [26] Despite research that indicates that benefit of coping flexibility and use of emotion-focused and problem-focused coping strategies, interventions have little sophistication in measuring changes in use of different coping strategies.

In this study, problem-focused coping used alone reduced psychological health in both boys and girls and emotion-focused coping used with problem-focused coping reduced psychological symptoms and enhanced well-being. Therefore, interventions should seek to specifically enhance emotion-focused coping skills such as emotion regulation, cognitive distraction or social support seeking. Research suggests that young children can be taught emotion-focused coping skills and these skills would enhance their coping repertoires and ability to deal with stress [20]. These interventions could support use of emotion-focused strategies such as emotional calming, positive emotion expression and relaxation techniques.

Some school-based programs have implemented interventions aimed at teaching youth emotion-focused coping strategies. The Rochester Child Resilience Project taught 4-6th grade children coping strategies to deal with stress based on 12 sessions program curriculum [74]. Two of these sessions included curriculum aimed at teaching children the distinction between solvable and unsolvable problems and use of strategies to deal with unsolvable problems (redirecting energy towards other tasks). While most of the sessions focused on problem-focused strategies, the overall program reduced measures of anxiety [74]. The Rational-Emotive Education Intervention developed by Vernon was another school based intervention for youth grades 1–6 to teach emotion education, problem solving skills and decision making [75]. Children learned how to identify negative feelings and change their thoughts, how to express emotions in positive ways and how to identify irrational thoughts. This intervention was positively related to pro-social behavior. A study in a humanitarian setting with trauma affected youth in Israel found that a school based intervention to provide children with effective coping strategies was effective in reducing PTSD. This Coping Enhancement Protocol intervention was delivered by teachers and taught students techniques focused on emotion regulation, such as methods to regulate negative emotions, distracting thoughts and relaxation techniques (awareness, muscle tension, breathing) as [76]. Schools may be an optimal space to deliver coping skill interventions. School based interventions can engage youth within a normative context where youth feel safe.

Interventions should also seek to provide structured positive activities for youth to engage that address daily stressors such as payment of school fees and food for self and family. Animal husbandry microfinance interventions, such as RFR, can provide mentorship by adults to youth to raise and breed small animals such as a rabbit for nutrition as well as create an income source by selling the rabbits in the local market to help family and siblings pay for school fees or medication, for example. The intervention may support youth to not only reduce daily stressors and improve mental health but these types of mentored group interventions can also support peer relationships and relationships with community members, an important source of emotional support. Furthermore, animal husbandry interventions can help to alleviate poverty long-term, which can help support improved mental health of current and future generations.

To address the burden of psychological distress in conflict-affected youth, research has shown that trauma focused group cognitive behavioral therapy (TF-CBT) has been effective in treating youth mental health in conflict-affected contexts [77–79]. Our research suggests that one of the means by which TF-CBT therapy may be effective is that it is able to synergize emotion-focused (cognitive) and problem-focused (behavioral) strategies to exploit the benefits that are not necessarily received if one strategy is used on its own. Providing TF-CBT therapy may also support overall coping strategy flexibility and build the coping repertoire of youth. Previous studies indicate that greater use of effective coping skills can mediate the effects of negative stressors on development of mental health problems [20, 80]. TF-CBT has been delivered at the group level, which maximizes use of limited resources available and may simultaneously help to support social relationships.

This research also indicates that girls and boy’s use of coping strategies may have different associations with mental health and well-being measures. While interventions may not need to target boys and girls separately, the impact of these interventions may differ by sex. Understanding how additional factors external to the individual is critical in designing effective interventions. In this study, individual coping strategies explain a relatively small amount of variance in outcomes, and additional variables at the peer, family and community level are likely important to understanding relationships of those variables with coping strategies and to help explain more fully the variance in outcome measures.

## Limitations

The *R* ^2^ for outcomes indicates that coping strategy explains between 5.7 and 21.7 % of the variation in internalizing and externalizing problems and 6.9–16.7 % of the variation in well-being measures. While these *R* ^2^ values fail to explain a significant proportion of the variance, they are similar to *R* ^2^ indices reported by previous research [4, 55, 81, 82]. The *R* ^2^ values indicate that coping strategies alone explain a small proportion of the variance in internalizing and externalizing problems and well-being measures, indicating that other factors both internal and external to youths’ lives may be important to investigate.

The KidCope was not originally developed to meet the requirements of factor analysis, and furthermore, the binary yes/no response categories suitable for younger children introduced challenges to factor analysis. Though we added additional items to the original KidCope scale to better capture coping constructs, the small number of items representing subscales in the original were an additional challenge. Future confirmatory analysis of the four factor solution should consider adding additional item. For example, the faith-based coping strategy could benefit from greater detail such as the positive/negative religious coping strategies [68]. The Harvard Trauma Questionnaire used to collect information on youth exposure to potentially traumatic stress was a self-report scale, and verification of stress exposure by adult caregivers was not conducted. Future research may consider comparing youth self-report exposure to reports from caregivers to enhance validity.

Selecting villages that were operationally feasible is a limitation and results may not be generalizable to other rural communities if they differ from our sample (i.e. more isolated, different socioeconomic and livelihood characteristics, different infrastructure and availability of educational and community resources). Given that this study is nested within an intervention program with three intervention groups, it is possible that participation in an intervention group could bias results. However, we tested intervention group as a control variable in this study and these tests indicated that intervention group assignment did not significantly alter results. Given that the purpose of this study was not to evaluate the intervention, we excluded intervention group as a control to present a more parsimonious model. The cross-sectional design of this study did not allow for causal conclusions, that is, it is possible that there are reciprocal relationships occurring between stress, coping and mental health and well-being outcomes. For example, youth with high levels of internalizing and externalizing problems, may use particular coping strategies more and may be at greater risk for further traumatic stress. To better understand these complex relationships, longitudinal studies are necessary.

Results from this study may not be generalizable to other contexts, particularly contexts that where youth are not exposed to conflict-related stressors. The villages sampled in this study were rural and coping strategies in urban conflict-affected contexts may differ. Age and youth’s developmental stage may alter types of coping strategies employed in response to stress and coping strategy adaptation over time warrants further research.

## Conclusion

This study provides a context specific, in-depth analysis of coping strategies on mental health and well-being outcomes. Four types of coping strategies specific to this sample were included in the analysis, problem-focused, emotion-focused, avoidant and faith based strategies. Problem-focused coping was associated with increased internalizing and externalizing problems and reduced pro-social behaviors. Emotion-focused coping had positive mediating impact on self esteem in boys. Avoidant coping reduced internalizing problems in girls but not boys; use of avoidant coping in girls also increased self-esteem. Faith based coping increased self-esteem in girls and boys. The interaction effect of use of problem-focused coping with emotion-focused coping reduced internalizing problems in girls and externalizing problems in girls and boys, suggesting that coping flexibility or use of more than one strategy can be beneficial to mental health. These findings suggest that interventions should support use of multiple coping strategies take advantage of the synergistic effect on reducing internalizing and externalizing problems and promoting well-being. Interventions should support faith-based and emotion-focused strategies to improving well-being. Additional research is needed to consider how external factors at the peer, family and community level moderate these findings.

## Abbreviations

DRC, Democratic Republic of Congo; PAIDEK, Programme d'Appui aux Initiatives Economiques; PFP, pigs for peace; RFR, rabbits for resilience
